# Modern flow chemistry – prospect and advantage

**DOI:** 10.3762/bjoc.19.3

**Published:** 2023-01-06

**Authors:** Philipp Heretsch

**Affiliations:** 1 Institute of Organic Chemistry, Leibniz Universität Hannover, Schneiderberg 1B, 30167 Hannover, Germany

**Keywords:** flow chemistry, method development, reactor design

Organic chemistry has shaped modern society by fulfilling the basic needs for pharmaceuticals, agrochemicals, fragrances, and many more. Implementation of new and innovative technologies has played a vital role in this mission and has contributed to the opening of new research areas and to pushing the frontiers of existing ones. Among these new technologies, continuous flow chemistry has stepped on the stage in the last decades [1]. Originating from the petrochemical industry, where it enabled high productivity and scalability even for the most standard processes of heating, cracking, and refining of crude oil to bulk chemicals [2], it has since entered the production of pharmaceuticals and other fine chemicals. This has again led to improved scalability, higher purity of products, and eventually decreased manufacturing costs.

From the undisputed role of continuous flow chemistry for process chemists, the advent of this technology in academic research laboratories, especially for method development and natural product synthesis programs [3], has revealed some inadequacies, particularly in view of the equipment and procedures available. These limitations have been slowly overcome with many creative but sometimes highly “academic” solutions.

Thus, recent years witnessed a steady increase in the application of continuous flow technology for academic research, leading to an expansion of synthetic options and generally more sustainable operations. Among the many advantages of performing organic reactions in continuous flow, enhanced heat-, mass- and photon transfer, an improved safety profile, broad scalability, and higher sustainability are the most prevalent ones.

To provide examples and explanation for these claims, “flash chemistry”, a term coined by late Yoshida [4] for reactions at the diffusion limit, i.e., reactions completed within milliseconds with proper mixing, showcases the fast heat- and mass transfer of continuous flow reactors. The generation of organolithium species in the presence of carbonyl compounds and their reaction has been facilitated by the extremely fast mixing of reagents and almost instantaneous heat transfer (i.e., cooling) in specifically designed microreactors [5].

Analogously, significantly increased photon transfer in flow reactors has been exploited. Where the molar attenuation coefficient is high, such as in many important photoredox catalysts, most of the irradiation is already absorbed within a thin layer of a few millimeters. Thus, in batch reactors the vast volume of the solution is not irradiated, and the reaction can only take place in the outermost layer [6–7]. This results in an extended reaction time when performing such reactions in bulk and may even lead to increased side reactions. Moving such operations into tubular flow reactors with a small diameter can both accelerate the transformations as well as lead to significantly less side products by continuously removing the products from the irradiation source.

As an example, Noël and co-workers performed efficient irradiation in flow for the C(sp^3^)–H functionalization of gaseous hydrocarbons [8], wherein photoexcited decatungstate was employed. Decatungstate is an efficient and versatile hydrogen atom transfer (HAT) catalyst with a growing number of applications. The use of decatungstate in a continuous flow setup led to shorter reaction times, increased scalability, and improved safety with pressurized gaseous alkanes employed in the abovementioned transformations.

Enhanced safety profiles of continuous flow reactors have been widely appreciated in industrial laboratories, while hazardous reactions still tend to be addressed subordinately or are even marginalized in academia [9]. The comparably small dimensions of flow reactors enable explosive, toxic, or otherwise dangerous reactions and reagents to be accumulated only to a much lesser degree, especially when scaling up.

This virtue has been exploited in process chemistry, where in the manufacturing of HIV protease inhibitor nelfinavir mesylate, diazomethane was an inevitable necessity. By moving the generation of this toxic and explosive reagent into a continuous process with manageable amounts present at any given point and continuously stripping the chemical with a stream of nitrogen, safety protocols could be met [10].

Among the disadvantages when moving from batch reactions to a continuous flow regime, dispersion phenomena play a detrimental role. These gradient effects occur when a stream of reagent is introduced into the reactor by pushing it with pure solvent. The reagents with well-defined concentration then leach into the solvent slug, leading to ill-defined stoichiometry and decrease in yield. While this axial dispersion is dependent on flow speed and residence time, only the central part of the reaction stream is under so-called steady-state conditions. To zero out these effects when a process yield is to be determined, pre- and postrun fractions are discarded [6], leading to loss of reagent and substrate as well as to increased waste. This is even less tolerable when small quantities of precious intermediates from multistep routes are to be employed, as is typically the case in projects of the pharmaceutical industry and academia.

A possible solution to this general problem was reported by Jensen and co-workers, who introduced segmented gas–liquid flow by mixing the reagent stream with an inert carrier gas, forming liquid and gaseous slugs moving through the reactor, and thus insolating liquid compartments from leaching into one another [11]. This concept has been adapted and further refined by Gilmore and co-workers, introducing automated multistep synthesizers [12], as well as Heretsch et al. in a setup optimized for natural products synthesis and late-stage manipulations [13].

To address customized reactor solutions from an engineering perspective, the advent of computer-aided design (CAD) in combination with widely available 3D printing has become a preferred choice. Prototyping aided by these technologies has significantly accelerated the development of novel flow reactor designs [14]. The challenging properties of organic solvents for standard polymers used in commercial 3D printers remain a drawback, while industrial 3D printers remain the much more expensive option. Reactors or other laboratory ware printed in polypropylene are restricted in use to only a few organic solvents or even to a single application, rendering this technology rather a supportive tool than a general solution [15]. Still, having this option available significantly lowers the barrier of inventing novel technological solutions and allows for high-throughput optimization in reactor design. Since different types of reactions typically call for an optimized or even specifically designed reactor built, the modularity of flow reactor is vital for quick reconfiguration and switching between different reactions.

This argument is particularly true when natural product synthesis is to be performed in continuous flow. The need for flexible and modular reactors that address the different demands associated with total synthesis poses particular challenges, among them performing fast reactions at low temperature, slow reactions at elevated temperature, reactions involving reactive gases under pressure, and photochemical reactions. Besides, also scalability is a major prerequisite in these synthetic endeavors, with reactions routinely being performed on a decagram scale in the early stages of a route and on a milligram scale at the end of a sequence. Designing modular reactors that meet these demands will help to overcome existing reservations for continuous flow in academia [13].

As guest editor of this thematic issue, I would like to express my gratitude to all authors for their excellent contributions. I thank the referees for providing their expertise and time and the whole team at the *Beilstein Journal of Organic Chemistry* for their professional support.

Philipp Heretsch

Hannover, December 2022
